# Comparison of Therapeutic Effects of Laparoscopic and Open Operation for Congenital Choledochal Cysts in Adults

**DOI:** 10.1155/2014/670260

**Published:** 2014-02-25

**Authors:** Yuan Liu, Xu Yao, Shuqiang Li, Wenhan Liu, Lei Liu, Jingang Liu

**Affiliations:** Department of General Surgery, Shengjing Hospital of China Medical University, Shenyang City 110004, China

## Abstract

*Background*. Laparoscopic cyst excision and Roux-en-Y hepaticojejunostomy for treating congenital choledochal cysts (CCCs) have proved to be efficacious in children. Its safety and efficacy in adult patients remain unknown. The purpose of this study was to determine whether the laparoscopic procedure was feasible and safe in adult patients. *Methods*. We reviewed 35 patients who underwent laparoscopic operation (laparoscopic group) and 39 patients who underwent an open procedure (open group). The operative time, intraoperative blood loss, time until bowel motion recovery, duration of drainage, postoperative stay, time until resumption of diet, postoperative complications, and perioperative laboratory values were recorded and analyzed in both groups. *Results*. The operative time was longer in the laparoscopic group and decreased significantly with accumulating surgical experience (*P* < 0.01). The mean intraoperative blood loss was significantly lower in the laparoscopic group (*P* < 0.01). The time until bowel peristalsis recovery, time until resumption of diet, abdominal drainage, and postoperative stay were significantly shorter in the laparoscopic group (*P* < 0.01). The postoperative complication rate was not higher in the laparoscopic group than in the open group (*P* > 0.05). *Conclusions*. Laparoscopic cyst excision and hepaticojejunostomy are a feasible, effective, and safe method for treating CCCs in adult patients.

## 1. Introduction

**Figure 1:**
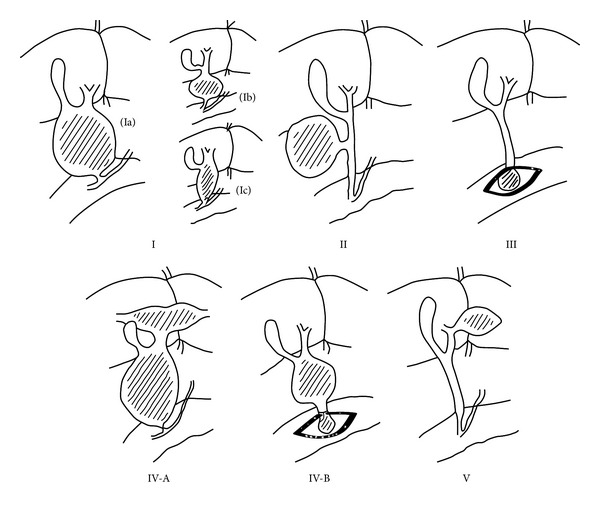
CCC classification: type Ia, cystic dilatation of the common bile duct with PBM; type Ib, focal segmental dilatation without PBM; type Ic, fusiform dilatation of the entire extrahepatic bile duct with PBM; type II, diverticular dilation of the common bile duct without PBM; type III, dilatation of the intraduodenal segment of the common bile duct (choledochocele) without PBM; type IVA, combined dilatations of intrahepatic and extrahepatic bile ducts, usually accompanied by PBM; type IVB, multiple dilatations of extrahepatic bile duct, PBM is uncertain; type V, cystic dilatations of the intrahepatic bile ducts (Caroli's disease) without PBM.

Congenital choledochal cyst (CCC) is a rare disease of the biliary tree, characterized by either isolated or combined dilatations of the extra- and intrahepatic bile ducts. CCCs occur most frequently in Asian and female populations [1, 2]. The etiology of CCCs remains unclear, although many theories have been put forth. Pancreaticobiliary maljunction (PBM) is observed in most cases and is considered the most important etiology of CCCs. PBM was first reported by Kozumi and Kodama [3] in 1916. According to the diagnostic criteria issued by the Japanese Study Group on PBM, PBM is defined as a union of the pancreatic and biliary ducts outside the duodenal wall [4]. The clinical presentation and management depend on the CCC type. The most common complication is cystolithiasis (49%) followed by cholangitis (32%), acute pancreatitis (10%), hepatolithiasis (7%), malignancy (3%), portal hypertension (2%), and chronic pancreatitis (2%) [5]. Cholangiocarcinoma is the most serious and dangerous complication. Different CCC classifications have been described in the literature. Todani's classification [6] dividing CCC into five types is the most useful in clinical practice (Figure 1). Early diagnosis and appropriate treatment are very important, because CCCs are associated with a risk of carcinogenesis, which increases with age. The diagnosis of CCCs depends on imaging investigations such as ultrasonography, computed tomography (CT), endoscopic retrograde cholangiopancreatography, and magnetic resonance cholangiopancreatography. Currently, prenatal diagnosis using ultrasonography is possible. In most patients, total cyst excision with Roux-en-Y hepaticojejunostomy is the treatment of choice [7–9]. Surgical treatment of CCCs is associated with a high success rate and low morbidity and mortality. With improvements in laparoscopic surgical skill, some pediatric surgeons have successfully carried out this procedure laparoscopically [7, 10–13].

Although most CCCs are diagnosed in the first decade of life [14–17], a recent series has suggested that the number of adults presenting with choledochal cyst disease is increasing [18]. Moreover, its presentation in adults appears to differ from that in children [19]. We recently began to use a total laparoscopic technique to treat adult patients with CCCs. Here, we compare the therapeutic effects and postoperative complications between the laparoscopic technique and open surgery in order to determine the feasibility and safety of the laparoscopic operation in adults with CCCs.

## 2. Materials and Methods

We reviewed the cases of adult CCC patients who underwent laparoscopic cyst excision and Roux-en-Y hepaticojejunostomy between May 2007 and November 2011. The control group consisted of CCC patients who underwent open cyst excision and hepaticojejunostomy by the same surgeon between December 2002 and April 2007. All the patients had no severe complications such as diffuse peritonitis caused by cyst rupture, cholestatic liver cirrhosis, or malignant change before operation. The study was approved by the ethics committee of Shengjing Hospital of China Medical University. The detailed clinical data of the subjects are shown in Table 1.

### 2.1. Laparoscopic Cyst Excision and Roux-en-Y Hepaticojejunostomy Techniques

**Figure 2:**
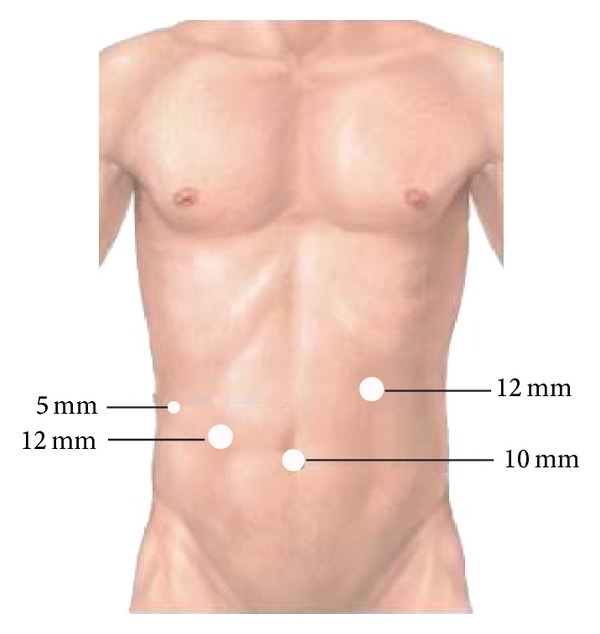
Sites and sizes of the trocar ports.

**Figure 3:**
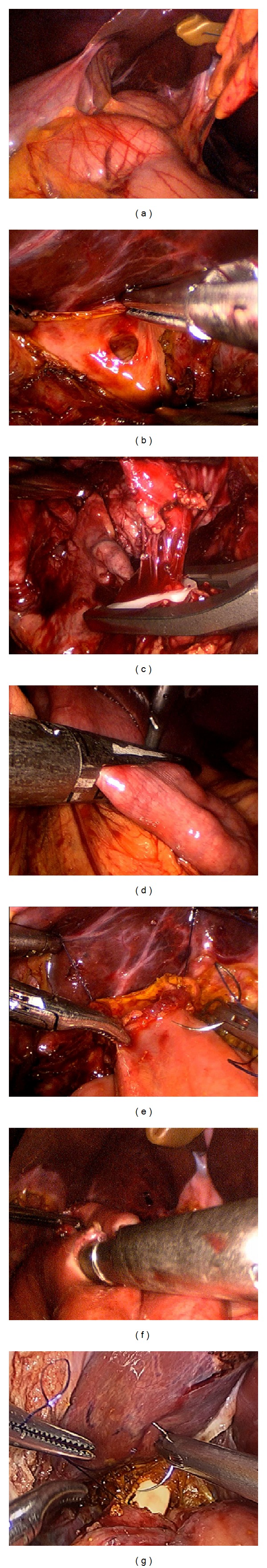
Laparoscopic cyst excision and Roux-en-Y hepaticojejunostomy. (a) Suspension of the left hepatic lobe. (b) A trumpet terminal was left after the transection of the common bile duct. (c) Clipping the distal narrow part of the cyst. (d) Amputation of the jejunum using an Endo-GIA linear stapler. (e) Hepaticojejunostomy with a running suture. (f) Jejunojejunostomy using an Endo-GIA linear stapler. (g) An intrabiliary stent tube was then placed.

Preoperative preparation included cleaning and disinfection of the umbilical region, placing a gastrointestinal decompression tube and a urinary catheter, and intravenous injection of prophylactic antibiotics. Under general anesthesia, the patients were placed in the reverse Trendelenburg position with abduction of both lower extremities. The operating surgeon stood between the patient's legs; two assistants stood on both sides of the patient. The monitor was placed on the cranial side of the patient. Carbon dioxide pneumoperitoneum was established, and the pressure was maintained at 13–15 mm Hg. A 10 mm 30° laparoscope was inserted through a 10 mm umbilical port. Three additional trocars then were inserted, as shown in Figure 2. After general exploration of the abdominal cavity, the left hepatic lobe was suspended for perfect visualization of the hepatic hilar region (Figure 3(a)). Next, a fundus-first cholecystectomy was performed. The cystic duct should be preserved for en bloc excision and traction. The anterior wall of the choledochal cyst was opened to depress the cyst and remove any calculi. The cyst was transected 0.5–1.0 cm beneath the normal common hepatic duct in order to create a trumpet-shaped terminal (Figure 3(b)). The cyst was dissected from its proximal to distal end, using a Harmonic scalpel. When the narrow, distal part of the cyst was exposed, it was clipped with a Hem-o-lok (Figure 3(c)), and the entire cyst was removed. The jejunum was amputated 15 cm distal to the Treitz ligament, using an Endo-GIA linear stapler (Figure 3(d)), and the mesentery of the small intestine was cut using LigaSure. The Roux limp of the jejunum was pulled to the hepatic hilum in an antecolic or retrocolic manner, and a hepaticojejunostomy was created in an end-to-side fashion by using the running suture technique with 4-0 absorbable sutures (Figure 3(e)). A side-to-side jejunojejunostomy was then created at a level 45 cm distal to the hepaticojejunostomy, using the Endo-GIA linear stapler (Figure 3(f)) and the continuous suture technique with 3-0 absorbable sutures. If the diameter of the anastomotic port was shorter than 0.8 cm, an intrabiliary stent tube was placed (Figure 3(g)). The skin ports were closed after the placement of a drain tube in the subhepatic space.

### 2.2. Open Cyst Excision and Roux-en-Y Hepaticojejunostomy Techniques

**Figure 4:**
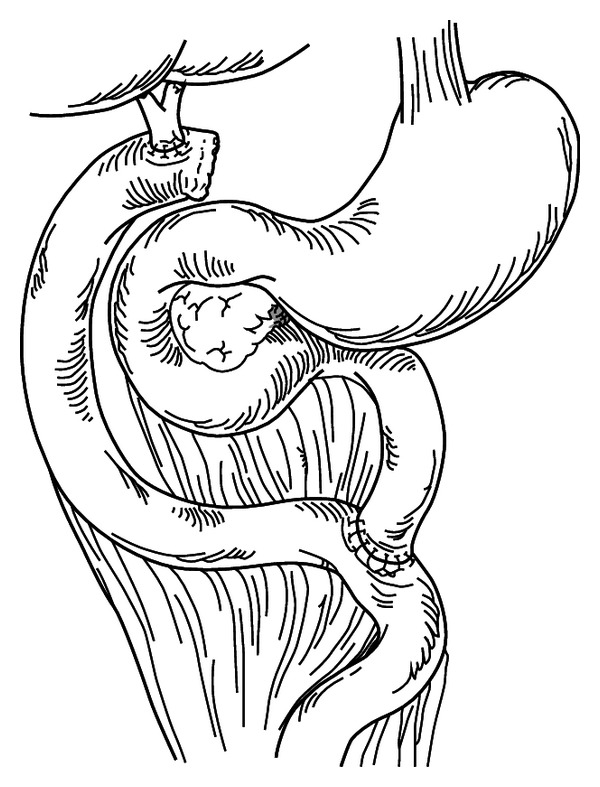
Open cyst excision and Roux-en-Y hepaticojejunostomy.

For the open approach, a right transrectus incision was selected. The procedure was accomplished in a similar fashion to the laparoscopic approach and mainly included gallbladder and cyst excision, hepaticojejunostomy, and jejunojejunostomy. The hepaticojejunostomy was performed with 4-0 absorbable sutures in a retrocolic manner. The jejunojejunostomy was created using a circular stapler in an end-to-side manner (Figure 4).

### 2.3. Discharge Criteria

The discharge criteria in both groups were as follows: (1) no abdominal pain or distension, (2) no fever, (3) no evidence of bile leakage, (4) no residual intraperitoneal fluid detected on postoperative CT, and (5) no indisposition after commencement of a semifluid diet per os.

### 2.4. Observation Indices

The operative time, intraoperative blood loss, time until bowel motion recovery, duration of drainage, hospital stay, time until resumption of diet, postoperative complications, and perioperative laboratory values were recorded and analyzed.

### 2.5. Followup

All patients were followed up at 3, 6, and 12 months postoperatively and at 6-month intervals thereafter. Physical examination, abdominal CT scanning and laboratory tests were performed at each visit. Upper gastrointestinal contrast examinations were performed if reflux cholangitis was suspected.

### 2.6. Statistical Analysis

Data were analyzed with the SPSS 13.0 package. The Student's *t*-test was used to compare the operative time, intraoperative blood loss, time until bowel motion recovery, duration of drainage, postoperative hospital stay, time until resumption of diet, and pre- and postoperative laboratory values between the two groups. The chi-square test was used to compare morbidity due to postoperative complications between the two groups. Paired *t*-tests were used to compare perioperative laboratory values in both groups. A level of *P* < 0.05 was considered statistically significant.

## 3. Results

Thirty-five CCC patients who underwent laparoscopic cyst excision and hepaticojejunostomy and were followed up for no less than 12 months were recruited. Thirty-nine CCC patients who underwent an open procedure were recruited as the control group. The median follow-up periods for the laparoscopic and open groups were 26 months (range, 12–54 months) and 98 months (range, 66–120 months), respectively.

### 3.1. Demographic Data and Clinical Manifestations

The demographic data and clinical manifestations have been summarized in Tables 1 and 2, respectively. The mean age did not significantly differ between the two groups (24.2 ± 8.3 years versus 26.7 ± 6.9 years, *P* > 0.05). The mean diameter of the common bile duct and the PBM rate also did not significantly differ between the two groups (*P* > 0.05). The main clinical symptoms included abdominal pain, abdominal mass, jaundice, fever, and/or pancreatitis. There was no significant difference between the two groups in the rate of symptomatic cases (85.7% versus 84.6%, *P* > 0.05).

### 3.2. Operative Time and Intraoperative Blood Loss

**Figure 5:**
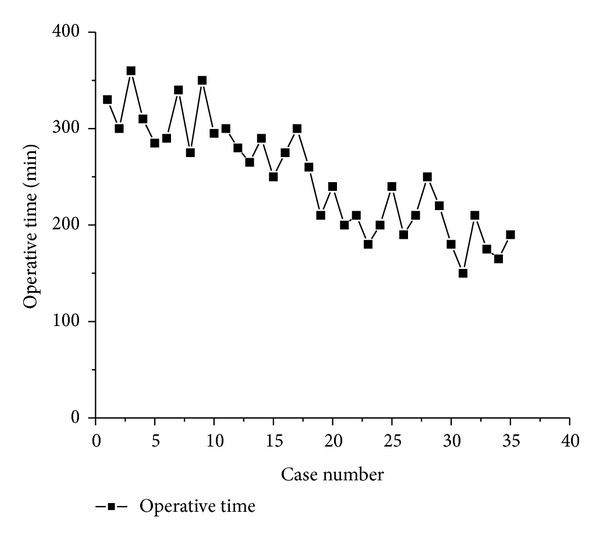
Operative times showed a tendency to decrease as the number of cases accumulated.

The mean operative time in the laparoscopic group was significantly longer than that in the open group (249 ± 58 min versus 132 ± 15 min, *P* < 0.01). The operative time in the laparoscopic group tended to decrease with accumulation of operating experience (Figure 5). Of interest, the mean operative time decreased significantly after the first 20 cases (290 ± 37 min versus 198 ± 27 min, *P* < 0.01). The mean intraoperative blood loss in the laparoscopic group was significantly less than that in the open group (72 ± 26 mL versus 174 ± 51 mL, *P* < 0.01; Table 3).

### 3.3. Postoperative Observations

The time until recovery of bowel peristalsis and the time until resumption of diet were significantly shorter in the laparoscopic group than in the open group (*P* < 0.01; Table 3). The duration of drainage was also significantly shorter in the laparoscopic group than in the open group (76 ± 24 h versus 103 ± 31 h, *P* < 0.05; Table 3). The mean postoperative stay was 6.2 ± 1.3 days in the laparoscopic group and 9.8 ± 0.8 days in the open group; the difference was statistically significant (*P* < 0.01; Table 3). Postoperative liver-function tests and serum amylase levels were improved in both groups (*P* < 0.01; Table 4).

### 3.4. Postoperative Complications

In total, six patients (17.1%) in the laparoscopic group and eight patients (20.5%) in the open group developed postoperative complications. This difference was not statistically significant (*P* > 0.05; Table 5). Similarly, no significant difference in the rate of postoperative biliary complications was observed between the two groups; however, this rate was slightly lower in the laparoscopic group (8.6% versus 12.8%, *P* > 0.05).

Most patients recovered with conservative management after surgery. Reoperation was necessary for two patients in the open group. One of these patients developed hepaticojejunostomy stenosis, which was treated by reforming the stoma through a laparotomy. The other patient underwent reoperation because of intrahepatic stone formation. There were no deaths in either group.

## 4. Discussion

Compared with children, adult patients have very different clinical manifestations of CCCs. In children, the main symptoms of CCCs are abdominal pain, mass, and/or jaundice. However, with aging, the manifestations of CCCs include biliary calculi, pancreatitis, and biliary tract cancer, including gallbladder cancer [20]. Once CCC has been diagnosed, early surgical treatment should be adopted to prevent recurrent episodes of cholangitis, which can lead to liver cirrhosis, carcinogenesis or cyst rupture, and other serious complications. Laparoscopic CCC excision and Roux-en-Y hepaticojejunostomy were first carried out by pediatric surgeons. Since Farello et al. first reported laparoscopic-assisted cyst excision and hepaticojejunostomy in 1995 [7]; reports about this technique for the treatment of CCC have gradually increased [21–23]. Moreover, the prognosis after this technique was satisfactory. However, the vast majority of these studies were carried out by pediatric surgeons. Few reports are available about laparoscopic surgery in adult CCC patients, in whom the feasibility and safety of this technique should be evaluated.

Compared with the open operation, total laparoscopic cyst incision and Roux-en-Y hepaticojejunostomy have a longer operative time. The laparoscopic procedure requires highly skilled manipulation as well as proficient collaboration between the surgeon and the assistants. During the early period after the introduction of the laparoscopic operation, surgeons lacked laparoscopic experience, and, understandably, the operative time for the laparoscopic procedure was significantly longer than that for the open procedure. Some experienced pediatric surgeons have reported that the difference in operative time between the laparoscopic technique and open procedure is not significant [20, 24]. In our study, the operative time in the laparoscopic group showed a declining tendency in pace with the accumulation of operative experience. Specifically, the operative time in the laparoscopic group significantly decreased (290.3 min versus 193.3 min) after the first 20 cases. As the learning curve progresses, we estimate that the operative time of the laparoscopic procedure will gradually approach that of open surgery.

Except for the longer operative time, laparoscopic surgery was more advantageous than open surgery. Due to the magnified field of vision during laparoscopy, the tiny blood vessels surrounding the cyst wall could be clearly seen. Besides, the hemostatic function of the Harmonic knife and LigaSure was effective and reliable. Therefore, the intraoperative blood loss in the laparoscopic group was significantly lower than that in the open group. Furthermore, because of the minimal wounds, the postoperative pain was slight. These patients willingly and easily followed the instructions for postoperative care, such as cough and early ambulation. Hence, the patients in the laparoscopic group recovered sooner and were discharged earlier.

The reported complications of the laparoscopic operation include bile leakage, respiratory tract infection, obstruction of the biliary limb, and incisional hernia of the trocar site [25]. The postoperative complications in the laparoscopic group were significantly decreased [11, 20, 26–32]. Bile leakage is the most common complication of surgery for choledochal cyst. In the initial stage, the rate of bile leakage after open operation was reported to be 5.8%–7.3% [9, 16]. Similar to the open operation, laparoscopic cyst excision and hepaticojejunostomy have recently been associated with a bile leakage rate of 1.6%–8.1% [12, 23, 29, 33]. In our study, more than 50% of the postoperative complications in the two groups were biliary complications, such as bile leakage, anastomotic stenosis, intrahepatic stone formation, and reflux cholangitis. Although there were no significant differences in the rate of total or biliary postoperative complications between the two groups, we predict that the complication rate will tend to decrease with accumulation of laparoscopic experience.

The “Y” shape jejunojejunostomy has been accomplished extracorporally by most surgeons through enlarging the umbilical incision [12, 30, 33]. We accomplished this procedure intraabdominally by using Endo-GIA and laparoscopic running sutures. The incision was cosmetic, and enlargement of the incision was avoided.

The prospective efficacy of total laparoscopic cyst excision and Roux-en-Y hepaticojejunostomy needs to be further investigated. According to the clinical experience in the last decade, the risk of postoperative complications has not increased due to laparoscopic operations [26, 34–37]. Although the operative time in the laparoscopic group was 1-2 h longer than that in the open group, the advantages of the minimally invasive surgery, such as small abdominal incision, obscure scar, less postoperative pain, fast recovery of bowel peristalsis, and fewer intraabdominal adhesions, cannot be ignored. Laparoscopic surgery will be the ideal choice for the treatment of CCCs in adults.

## Figures and Tables

**Table 1:** Demographic features of the laparoscopic group versus open group.

	Laparoscopic group *n* = 35	Open group *n* = 39	*P* value
Age: years (range)	24.2 ± 8.3 (15~46)	26.7 ± 6.9 (17~42)	>0.05
Sex (male/female)	6/29	8/31	
Todani's type			
Ia	18	21	
Ic	14	13	
IVA	3	5	
Cyst diameter: cm (range)	6.6 ± 1.8 (3.5–14.5)	7.3 ± 2.4 (4.0–16.0)	>0.05
PBM rate (*n*)	80.0% (28)	76.9% (30)	>0.05

**Table 2:** Clinical manifestations of laparoscopic group versus open group.

	Laparoscopic group *n* = 35	Open group *n* = 39	*P* value
Symptomatic rate (*n*)	85.7% (30)	84.6% (33)	>0.05
Abdominal pain	80.0% (28)	66.7% (26)	
Abdominal mass	8.6% (3)	5.1% (2)	
Biliary stone	31.4% (11)	28.6% (10)	
Jaundice	25.7% (9)	23.1% (9)	
Fever	11.4% (4)	15.4% (6)	
Pancreatitis	11.4% (4)	7.7% (3)	
None (*n*)	14.3% (5)	15.4% (6)	

**Table 3:** Perioperative observation items of laparoscopic group versus open group.

	Laparoscopic group (*n* = 35)	Open group (*n* = 39)	*P* value
Operative time: min (range)	249 ± 58 (150~360)	132 ± 15 (110~160)	<0.01
Intraoperative blood loss: mL (range)	72 ± 26 (40~210)	174 ± 51 (80~550)	<0.01
Duration of bowel peristalsis recovery: h (range)	60 ± 13 (36~108)	102 ± 11 (72~120)	<0.01
Resumption of diet: days (range)	72 ± 16 (48~108)	108 ± 9 (84~120)	<0.05
Duration of drainage: h (range)	76 ± 24 (72~192)	103 ± 31 (72~264)	<0.05
Postoperative hospital stay: d (range)	6.2 ± 1.3 (5~9)	9.8 ± 0.8 (8~13)	<0.05

**Table 4:** Perioperative laboratory results of laparoscopic group versus open group.

	Laparoscopic group (*n* = 35)	Open group (*n* = 39)	*P* value
ALT (U/L)			
Preoperative	165.6 ± 46.9	153 ± 54.7	>0.05
Postoperative	34.4 ± 16.8*	38.2 ± 15.3*	>0.05
AST (U/L)			
Preoperative	155.5 ± 50.2	148.4 ± 61.1	>0.05
Postoperative	28.5 ± 14.7*	33.7 ± 12.5*	>0.05
ALP (U/L)			
Preoperative	622.4 ± 77.5	588.3 ± 65.9	>0.05
Postoperative	144.2 ± 40.6*	167.0 ± 52.1*	>0.05
GGT (U/L)			
Preoperative	385.0 ± 60.4	401.7 ± 66.6	>0.05
Postoperative	50.6 ± 14.0*	55.3 ± 17.2*	>0.05
TBIL (*μ*mol/L)			
Preoperative	77.6 ± 20.3	80.5 ± 18.8	>0.05
Postoperative	14.4 ± 5.0*	15.3 ± 4.7*	>0.05
DBIL (*μ*mol/L)			
Preoperative	67.1 ± 17.1	69.4 ± 19.8	>0.05
Postoperative	7.8 ± 3.5*	8.2 ± 2.8*	>0.05
SAMY (U/L)			
Preoperative	344.9 ± 288.5	388.7 ± 301.6	>0.05
Postoperative	45.2 ± 22.3*	43.3 ± 25.8*	>0.05

Note: ALT: alanine transaminase, AST: aspartate aminotransferase, ALP: alkaline phosphatase, GGT: *γ*-glutamyl transpeptidase. TBIL: total bilirubin, DBIL: direct bilirubin, and SAMY: serum amylase.

*P < 0.01 compared with preoperative values.

**Table 5:** Postoperative complications of the laparoscopic group versus open group.

	Laparoscopic group (*n* = 35)	Open group (*n* = 39)	*P* value
Intraabdominal hemorrhage	1	0	
Gastrointestinal bleeding	0	1	
Pancreatic juice leakage	1	0	
Pancreatitis	0	0	
Surgical site infection	0	1	
Biliary limb obstruction	1	0	
Adhesive intestinal obstruction	0	1	
Biliary complications	3	5	>0.05
Self-limiting bile leakage	(2)	(2)	
Anastomotic stenosis	(0)	(1)	
Intrahepatic stone formation	(0)	(1)	
Refluxing cholangitis	(1)	(1)	

Total	6	8	>0.05
